# Effect of the Ongoing Severe Acute Respiratory Syndrome Coronavirus 2 (SARS-CoV-2) Pandemic on Dental Service Utilization in Patients With Comorbidities

**DOI:** 10.7759/cureus.39926

**Published:** 2023-06-03

**Authors:** Ozlem Akkemik, Aydan Acikgoz

**Affiliations:** 1 Dentomaxillofacial Radiology, Baris Medical Imaging Center, Izmir, TUR; 2 Dentomaxillofacial Radiology, Faculty of Dentistry, Yeni Yuzyil University, Istanbul, TUR

**Keywords:** dental practice, dentistry curriculum, comorbidities, pandemic, global healthcare systems

## Abstract

Background: The keystone of safe and effective patient management is to approach a patient with up-to-date medical information. Assessment of patients for their medical conditions has changed during the coronavirus disease 2019 (COVID-19) pandemic and the need for appropriate research infrastructure has increased. Considering an updated list of high-risk underlying conditions in the post-COVID-19 era, this study aimed to evaluate the utilization of dental services by patients with comorbidities during the severe acute respiratory syndrome coronavirus 2 (SARS-CoV-2) pandemic.

Methods: Data of patients with comorbidities seeking dental care at a dental school during the COVID-19 pandemic were retrospectively evaluated. Demographic variables (age, gender) and medical history of the participants were recorded. The patients were classified according to their diagnosis. Data were analyzed using descriptive statistics and Chi-square analysis. The significance level was determined at *α*=0.05.

Results: The study included data from 1067 patient visits between September 1, 2020 and November 1, 2021. Among these patients, 406 (38.1%) were males and 661 (61.9%) were females, with a mean age of 38.28 ± 14.36 years. Comorbidities were identified in 38.3% of the patients with predominance in females (74.1% n=303). Single comorbidity was observed in 28.1% while multi-morbidity was detected in 10.2% of the cohort. The most prevalent comorbidity was hypertension (9.7%), followed by diabetes (6.5%), thyroid disorders (5%), various psychological diseases (4.5%), COVID-19 infection (4.5%), and different allergies (4%). The presence of one or more co-morbidities was observed mostly in the 50-59 years age group.

Conclusions: The seeking of dental care among the adult population with comorbidities was high during the SARS-CoV-2 pandemic. It would be beneficial to develop a template for obtaining a medical history from patients by taking full account of the consequences of the pandemic. The dental profession needs to respond accordingly.

## Introduction

Coronaviruses are a group of related RNA viruses that cause diseases in mammals and other animals. Infection with coronaviruses differs significantly in risk, with some species of the virus being relatively harmless causing a cold, while others can give rise to diseases such as severe acute respiratory syndrome coronavirus (SARS-CoV), Middle East respiratory syndrome (MERS) and SARS-CoV-2, resulting in pandemics with high mortality [1]. SARS-CoV-2, the third coronavirus relevant to human infection that has been described to date, primarily causes lower respiratory tract symptoms with pulmonary complications. The disease was named coronavirus disease-2019 (COVID-19) and the infection is known to be transmitted through respiratory droplets or by direct contact. Therefore, dental activities were restricted only to emergencies during the first wave of the pandemic. Subsequently, the delivery of dental care was resumed, using the highest level of personal protective equipment [2].

Our knowledge about coronaviruses continues to expand. Meanwhile, individuals with pre-existing comorbidities including hypertension, diabetes mellitus (DM), cardiovascular disease (CVD), cerebrovascular disease, obesity, and chronic pulmonary disease have been reported to be relatively more vulnerable to COVID-19 [3]. In this context, the list of underlying medical conditions that may make a patient more vulnerable to COVID-19 is not exhaustive and should be updated as science advances. Similarly, patients seeking dental care should be evaluated through an updated list of underlying high-risk medical conditions to ensure safe and effective patient management. Dental service utilization may provide an opportunity for the screening of chronic disease. Several studies have examined the impact of COVID-19 on the dental profession and dentists, but to the best of our knowledge, none of the studies have addressed the utilization of dental clinics by patients with underlying medical conditions during the ongoing pandemic. Such data may be informative in understanding the dental care-seeking behavior of vulnerable populations and helping determine the need for effective interventions at a population level. Furthermore, monitoring adult dental patients with comorbidities can provide more comprehensive and updated data on underlying conditions in the adult patient population. Therefore, we used data from a Dental University to examine whether the presence of comorbidities was associated with decreased utilization of dental services during the COVID-19 pandemic. Findings from the current study may provide proof of concept of the benefits of innovative screening/care opportunities to better identify patients with comorbidities.

## Materials and methods

This observational cross-sectional study was approved by the Science, Social, and Non-Interventional Health Sciences Research Ethics Committee of the Yeni Yuzyil University of Medicine (06.12.21/No:2021/12-737). The research was conducted at the Faculty of Dentistry, Yeni Yuzyil University in Istanbul, Turkey. The study population consisted of patients who were admitted to the Department of Oral Diagnosis and Maxillofacial Radiology with various dental complaints between September 2020 and November 2021. Written informed consent was obtained from all participants. A total of 1067 adult patients aged 18 years and over were included in the study. Patients with incomplete anamnesis information were excluded from the study.

The anamnesis forms that were filled in by a specialist physician (A.A.) during the in-person interviews were recalled from the archives and evaluated retrospectively. The demographic data (gender and age) and presence of systemic diseases of the patients were recorded from each anamnesis form. Medical conditions were classified into 17 categories. If a patient had one systemic disease besides the dental complaint, he/she was classified in the comorbidity group. In case of more than one disease, he/she was included in the multi-morbidity group. Patients were divided into age groups with 10-year intervals: 18-29, 30-39, 40-49, 50-59, 60-69, and >70 years. Patients with cardiovascular diseases (CVDs), endocrine diseases, and respiratory diseases were further divided into multiple subgroups for more detailed evaluation. The patients were also queried for any SARS-CoV-2 infection in the past year. All findings were recorded in a spreadsheet (Microsoft Excel).

Statistical analysis of the data was performed with SPSS v.25.0 (SPSS Inc., Chicago, IL, USA). The differences in percentage and distribution of comorbidities in relation to age and gender were analyzed by the Chi-square test or Fisher’s exact test, where appropriate. Statistical significance was evaluated as p < 0.05.

## Results

Of the 1067 patients recruited to the current study, 661 (61.9%) were females while 406 (38.1%) were males. The mean age of female patients was 37.80 ± 14.23 years while the mean age of male patients was 39.06 ± 14.56 years and the mean age of the entire cohort was 38.28 ± 14.36 years. The difference in age distribution according to the gender of patients did not reach statistical significance (p > 0.05) (Table 1). The mean age of the patients without any medical condition (n= 658, 61.7%) was 34.2 years, while the mean age of patients with comorbidity or multimorbidity (n=409, 38.3%) was 44.9 years (Table 2). The distribution of comorbid diseases according to age groups was statistically significant (p < 0.05, Table 3). The comorbidities were significantly higher among females (n=30, 74.1%) and were most common for the age group of 50-59 years (n=97, 23.7%). Comorbid diseases were observed in 73.0% of female participants (n=219) and most commonly in the age group of 30-39 years (24.7%). Multimorbidity was found in 77.1% of females (n=84) and mostly in the age group of 50-59 years (31.2%) (p < 0.05).

**Table 1:** Distribution of patients according to age groups and gender (statistical significance p < 0.05).

Age groups	Female (n=661) n (%)	Male (n=406) n (%)	Total (n=1067) n (%)
18-29	216 (62.6%)	129 (37.4%)	345 (32.3%)
30-39	173 (65%)	93 (35.0%)	266 (24.9%)
40-49	123 (61.5%)	77 (38.5%)	200 (18.7%)
50-59	94 (59.5%)	64 (40.5%)	158 (14.8%)
60-69	38 (51.4%)	36 (48.6%)	74 (6.9%)
>70	17 (70.8%)	7 (29.2%)	24 (2.2%)

**Table 2:** Mean age of dental patients with systemic diseases according to gender. COPD, chronic obstructive pulmonary disease; CURI, chronic upper respiratory infection

Medical conditions	Age (females in years)	Age (males in years)	Age (total in years)
Cardiovascular disease	56.55	53.07	55.44
Hypertension	58.78	54.26	57.43
Cardiac disease	52.12	52.20	52.16
Dyslipidemia	58.46	61.25	59.12
Endocrine disorders	48.36	52.39	49.09
Type I diabetes	45.25	56.33	48.27
Type II diabetes	52.85	52.53	52.77
Thyroid disorders	45.22	53.00	45.51
Psychological disorders	41.21	40.93	41.13
Covid-19	31.21	39.43	33.60
Respiratory diseases	39.91	40.22	39.98
Asthma	40.20	41.17	40.36
COPD	49.00	-	49.00
CURI	39.00	31.00	34.43
Allergy	38.61	38.00	38.51
Musculoskeletal diseases	39.27	39.56	39.38
Cancer (past or present)	49.61	66.00	52.59
Gastrointestinal diseases	41.50	41.20	41.33
Hematological diseases	31.23	40.50	32.37
Neurological disorders	36.58	28.50	35.43
Autoimmune diseases	44.20	34.00	42.50
Renal diseases	40.60	33.00	39.33
Vitamin deficiencies	32.50	43.50	39.83
Infectious diseases	38.67	42.00	40.00
Autoinflammatory diseases	31.50	-	31.50
Liver diseases	64.00	-	64.00

**Table 3:** Age groups and distribution of systemic diseases (statistical significance p < 0.05).

	18-29 (n=345)	30-39 (n=266)	40-49 (n=200)	50-59 (n=158)	60-69 (n=74)	70+ (n=24)	Total (n=1067)
	n (%)	n (%)	n (%)	n (%)	n (%)	n (%)	n (%)
Comorbidity	63 (21.0%)	74 (24.7%)	58 (19.3%)	63 (21.0%)	30 (10.0%)	12 (4.0%)	300 (28.1%)
Multimorbidity	8 (7.3%)	9 (8.3%)	25 (22.9%)	34 (31.2%)	25 (22.9%)	8 (7.2%)	109 (10.2%)
Total	71 (17.4%)	83 (20.3%)	83 (20.3%)	97 (23.7%)	55 (13.4%)	20 (4.9%)	409 (38.39%)

The incidence of pre-existing medical conditions was found to be 38.3% (n=409) with 28.1% having comorbidity, and 10.2% having multimorbidity (Table 4). CVDs were the most common medical condition encountered in the current study population (12.3%, n=131). Among the various CVDs, hypertension was the most common (9.7%; n=104), followed by heart disease (n=32; 3.0%) and dyslipidemia (n=17; 1.6%). Although 67.9% of the CVDs patients were females, the difference in the incidence of CVDs as a function of gender did not reach statistical significance. However, the incidence of CVDs was significantly greater in older patients. CVDs, hypertension, and heart disease were observed most frequently in patients in the age group of 50-59 years with incident rates of 36.6%, 35.6% and 44.4%, respectively.

**Table 4:** Prevalence of dental patients with systemic diseases according to gender. COPD, chronic obstructive pulmonary disease; CURI, chronic upper respiratory infection

Medical condition	Female	Male	Total
Without systemic diseases	358 (54.4%)	300 (45.6%)	658 (61.7%)
With systemic diseases	303 (74.1%)	106 (25.9%)	409 (38.3%)
Comorbidity	219 (73%)	81 (27%)	300 (28.1%)
Multimorbidity	84(77.1%)	25 (22.9%)	109 (10.2%)
Cardiovascular disease	89 (67.9%)	42 (32.1%)	131 (12.3%)
Hypertension	73 (70.2%)	31 (29.8%)	104 (9.7%)
Cardiac disease	17 (53.1%)	15 (46.9%)	32(3.0%)
Dyslipidemia	13 (76.5%)	4 (33.5%)	17 (1.6%)
Endocrine disorders	105 (82.0%)	23 (18%)	128 (%12.0)
Type I diabetes	3 (27.3%)	8 (72.7%)	11 (1.0%)
Type II diabetes	52 (75.4%)	17 (24.6%)	69 (6.5%)
Thyroid disorders	51 (96.2%)	2 (3.8%)	53 (5.0%)
Psychological disorders	33 (68.8%)	15 (31.2%)	48 (4.5%)
Covid-19	34 (70.8%)	14 (29.2%)	48 (4.5%)
Respiratory diseases	35 (79.5%)	9 (20.5%)	44 (4.1%)
Asthma	30 (83.3%)	6 (16.7%)	36 (3.4%)
COPD	1	-	1 (0.1%)
CURI	4	3	7 (0.7%)
Allergy	36 (83.7%)	7 (16.3%)	43 (4%)
Musculoskeletal diseases	15 (62.5%)	9 (37.5%)	24 (2.2%)
Cancer (past or present)	18 (81.8%)	4 (18.2%)	22 (2.1%)
Gastrointestinal diseases	8 (44.4%)	10 (55.6%)	18 (1.7%)
Hematological diseases	13 (86.7%)	2 (13.3%)	15 (1.4%)
Neurological disorders	12 (85.7%)	2 (14.3%)	14 (1.3%)
Autoimmune diseases	10(83.3%)	2 (16.7%)	12 (1.1%)
Renal diseases	5 (83.3%)	1 (16.7%)	6 (0.6%)
Vitamin deficiencies	2 (33.3%)	4 (66.7%)	6 (0.6%)
Infectious diseases	3 (60.0%)	2 (40.0%)	5 (0.5%)
Autoinflammatory diseases	4 (0.4%)	-	4 (0.4%)
Liver diseases	2 (0.2%)	-	2 (0.2%)

Endocrine disorders (n=128; 12.0%) were the second most frequently detected medical condition and were again predominantly seen in female patients. Except for type I diabetes, a significant relationship was observed between endocrine diseases and age. Endocrine disease, type II diabetes, and thyroid disorders were found to be most common in patients belonging to the 50-59 years age group with incidence rates of 27.3%, 36%, and 22.6%, respectively.

Respiratory diseases (n=44; 4.1%) were also seen more commonly in females (n=35, 79.5%). The most common condition in this group of diseases was asthma (n=36, 3.4%) and its prevalence was significantly higher in females (n=30, 83.3%). Although the difference in the distribution of allergic diseases (n=43, 4.0%) as a function of age did not reach statistical significance, its distribution as a function of gender was found to be significant with females showing a higher prevalence (n=36, 83.7%). Cancer (n=22, 2.1%) was also detected in significantly more females (n=18, 81.8%) and more commonly in older patients (31.8%, 60-69 years group). The distribution of hematological diseases (n=15, 1.4%) as a function of gender reached statistical significance with hematological disorders being present mostly in female patients (n=13, 86.7%). The distribution of patients with asthma or psychological illnesses did not show any significant difference between the different age groups (p > 0.05).

Although 70.8% (n=34) of patients with a history of infection with SARS-CoV-2 were females, there was no statistically significant difference in the distribution of patients with a history of COVID-19 as a function of gender or age. Some 47.9% of the recovered COVID-19 patients were in the age group of 18-29 years, 18.8% were in the age group of 30-39 years, and 18.8% were in the age group of 40-49 years. It was determined that 97.3% of patients with systemic diseases did not have COVID-19. None of the patients with multimorbidity had COVID-19, while 3.7% of patients with comorbidity had COVID-19.

The prevalence of dyslipidemia, type 1 diabetes, chronic obstructive pulmonary disease (COPD), chronic upper respiratory infection (CURI), gastrointestinal (GI), hematological diseases, autoimmune diseases, neurological disorders, liver diseases, renal diseases, autoinflammatory or infectious diseases, and vitamin deficiencies was low in the cohort evaluated in the current study; therefore, the distribution of these diseases as a function of age could not be evaluated statistically.

The distribution of COPD and CURI, GI discomfort, psychological and autoimmune diseases, neurological diseases, disorders in the liver, kidney and musculoskeletal system, autoinflammatory and infectious diseases, and vitamin deficiency as a function of gender did not reach statistical significance (p > 0.05).

## Discussion

The vast number of studies reported during the COVID-19 pandemic reflects the high motivation of scientists to fill the gaps in clinical knowledge of various pathophysiological conditions. Data from the current cross-sectional study indicates that 38.3% of dental patients had an underlying medical condition with 28.1% having a comorbidity and 10.2% having multimorbidity (Table 5) [4-16]. This figure falls within the range reported in the literature (10%-69%). Among the 1076 dental patients evaluated in the current study, the most frequent comorbidities in the order of prevalence were hypertension, diabetes mellitus, thyroid diseases, psychological disorders, recovered COVID-19 infection, allergy, and others, respectively. However, evaluating the currently available literature, we determined a need for clinical studies on the impact of the pandemic on individuals with underlying disorders seeking dental care.

**Table 5:** Compilation of previously published studies showing the distribution of systemic diseases among dental patients. GIS, gastrointestinal disorder; CVD, cardiovascular disease; COPD, chronic obstructive pulmonary disease; BJD, bone/joint disease; DM, diabetes mellitus; ED, endocrine disorder; Derm. D, dermatological disorder; Dig. Dis., digestive diseases

Reference	Study group (n)	Age range years old	Gender ratio	Presence of diseases (%)	Top three most common diseases	Females with systemic diseases	Males with systemic diseases	Age in patients with systemic diseases
Smeets et al. (1998) [4]	29,424	7 age group (18-75+)	Female: 15,400 (52.3%) Men: 13.551 (46.1%)	28.2	Allergy (8.7%) CVD (6.8%) Hypertension (4.4%)	No significant difference in age between the male and female patients	No significant difference in age between the male and female patients	65-74 (23.9%) 75 or over (34.9%)
Jainkittivong et al. (2004) [5]	510	60-64 65-69 >70	Female: 310 (60.8%) Men: 200 (39.2%)	82.5	CVD (33.7%) BJD (32.4%) Allergy (18.2 %)	86.5%	76.5%	The incidence of medical conditions did not differ among the three age groups
Ilguy et al. (2005) [6]	13,527	8 age group (16-81+)	Female: 8267 (61%) Men: 5260 (39%)	35.6	Hypertension (12.8%) CVD (6.4%) Thyroid Disorders (5.3%)	No report total ratio	No report total ratio	No report on the total ratio
Radfar and Suresh (2007) [7]	1041	4 age group (18-80+)	Female: 533 (52.2%) Men: 488 (47.8%)	54	Hypertension (22%) DM (14%) Arthritis (13%)	58.9%	51.2%	60-79 the highest level of medical issues (64%)
Al-Bayaty et al. (2009) [8]	571	7 age group (15-70+)	Female: 360 (63%) Men: 211 (27%)	42	Hypertension (1.26%) DM (6.1%) Asthma (5.8%)	47%	33.4%	21-50 years old 67.4%
Dhanuthai (2009) [9]	58,317	1-95	No report total ratio	12.2	Allergy CVD ED	64.02%	35.98%	The majority in the fifth to the seventh decades (54.3%)
Bhateja (2012) [10]	36,729	No report total ratio	No report total ratio	1.02	CVD (57.87%) ED (35.73%) Respiratory diseases (7.47%)	46.93%	53.06%	50.04 ± 13.21
Maryam et al. (2015) [11]	1188	8-92	Female:760 (64%) Men: 428 (36%)	73.3	CVD (34.1%) DM (5.6%) Thyroid disorders (4.5%)	52%	24%	39.28 ± 15.65
Bozdemir et al. (2016) [12]	709	> 60	Female: 334 (47%) Men: 375 (53%)	90	CVD (65%) BJD ED	321 (45.3%)	317 (44.7%)	60-64 age groups (most systemic diseases)
Walia et al. (2017) [13]	5040	30-80	No report total ratio	26.5	Hypertension (13.8%) DM (8.29%) CVD (7.28%)	642 (48.05%).	694 (51.95%)	45.2 ± 11.6 majority of patients (54%) in the fourth to the sixth decades
Wadhwani et al. (2017) [14]	1000	<20 20-40 > 40	No report total ratio	22.1	Hypertension (7.6%) DM (4.9%) Derm. D. (4.6%)	No report total ratio	No report total ratio	<20 years: n= 12 20-40 years: n=39 >40 years: n= 170
Taghibakhsh et al. 2018 [15]	< 45 > 45	6270	Female: 50.7% Men: 49.3%	41.4	Hypertension (8.42%) Thyroid diseases (7.05%) Dig. Dis. (6.5%)	55.8%	49.3%	<45 years: 41.4% >45 years: 48,5%
Frydrych et al. (2020) [16]	7 age group 10 years apart (25-85+)	873	Male: 394 (45.1%) Female: 479 (54.9%)	86.3	CVD (37.9%) Allergy (32.3%) Mental health disorders (29.4%)	84.1%	88.8%	<25 years

The mean age of dental patients with comorbidities was similar to other studies published prior to the current pandemic, indicating that co-morbidities mainly affected the middle-aged and elderly (only one study showed the lack of any relationship between a pre-existing medical condition and age among dental school patients and may be considered to be exceptional) [7, 12, 14-15]. The incidence of chronic diseases is increasing worldwide due to an aging population and changing lifestyles. Changes in the immune system occur throughout life via two fundamental paths. One is the gradual deterioration of immune function defined as immunosenescence. The other, referred to as inflammaging, is a chronic increase in systemic inflammation via age-associated pro-inflammatory markers. According to epidemiological studies, this ineffective alert system is a risk factor for multimorbidities. Furthermore, the presence of comorbidities or a general deficiency of resilience in the elderly are the other possible explanations for why older individuals are more vulnerable to COVID-19 [17-18].

Except for one study [7], most studies suggest that a greater proportion of patients who applied to the dental outpatient clinic in the pre-pandemic period were females [8, 11-12, 15]. Supporting these data, the current study also found that predominantly female patients, both with and without comorbidities, visited dental clinics during the pandemic period as well. Differences in the access of women and men to dental care or other health conditions have already been established. There are clear biological motives for such differences. No doubt, the use of medical services during the COVID-19 pandemic has changed based on the perceptions and attitudes of patients, which should be addressed in further studies.

COVID-19 is now acknowledged as a multisystem disease with varied manifestations. Pathophysiologically similar to SARS-CoV, SARS-CoV-2 binds to the angiotensin-converting enzyme 2 (ACE2) receptor of host cells for cellular entry. The ACE2 receptor is highly expressed in the endothelium, lungs, heart, kidneys, and intestines. ACE2 is a major regulator of the renin-angiotensin-aldosterone system. Certain comorbidities such as hypertension, diabetes mellitus, and CVDs are known to increase ACE2 levels, which may exacerbate the severity of COVID-19 and susceptibility to the disease [1]. The impairment of the endothelium, which is another target organ, plays a key role in thromboembolic events. Therefore, patients with pre-existing CVDs should be determined and treated as a priority.

Similar to previous studies, data collected during the COVID-19 pandemic found CVDs to be the most common medical condition among dental patients [4-5, 8-10]. Although there is a strong gender bias with males showing high preponderance in the development of CVDs, the effect of gender on the development of CVDs among dental patients was not found to be statistically significant in the current work. Additionally, some studies have reported an increased prevalence of CVDs in females [9, 12]. When the data were analyzed according to age groups, a correlation was found between increasing age and the incidence of CVDs, in accordance with previous reports [11-12]. The data from dental patients in the pre-pandemic period indicated hypertension as the most frequently recorded medical condition, consistent with the findings of the current study carried out during the pandemic [7-8, 11-12, 14].

Dyslipidemia is considered to be a comorbid condition for the development of atherosclerotic CVDs. Dyslipidemia was shown to potentially increase the mortality and severity of COVID-19. However, the question remains whether the association between the severity of COVID-19 and dyslipidemia may be due to other comorbidities accompanying dyslipidemia, rather than dyslipidemia itself [3, 19]. Dyslipidemia was detected in 1.2% of the present study population whereas Radfar and Suresh found dyslipidemia to be the fourth most common medical condition in a pre-pandemic study among the dental population [7].

Diabetes is a complex chronic disease characterized by a dysregulation in plasma glucose levels. Patients with diabetes are more susceptible to infections. Any respiratory infection is known to be associated with the development of temporary insulin resistance. Even when diabetes is well controlled, diabetic patients present with long-term manifestations of the underlying disease at dental clinics [20]. Long-term effects of diabetes include the development of vascular complications and atherosclerosis. In diabetic patients, stress from dental procedures may create potential conditions for medical emergencies. In accordance with previous reports, we determined that diabetes was one of the most common comorbidities among patients seeking dental treatment during the pandemic period [7-8, 11-12, 14].

In the current study, we found a high proportion of dental patients with thyroid disorders. Very few reliable studies reported so far have evaluated thyroid diseases among dental patients [11, 15]. The presence of thyroid dysfunction or symptoms of hypo- or hyperthyroidism among dental patients can preclude dental treatments. Various studies have evaluated several potential effects of SARS-CoV-2 infection on thyroid function without determining the actual impact of COVID-19 on the management of thyroid patients. Infections are known to provoke autoimmune reactions and the role of viral infections in the initiation of autoimmune diseases has been established. A cross-talk between autoimmune diseases and COVID‐19 has also been reported [21]. Autoimmune diseases (e.g., rheumatoid arthritis, multiple sclerosis, systemic lupus erythematosus, rheumatoid arthritis, Hashimoto’s thyroiditis) can affect the adaptive immune system, while autoinflammatory diseases (e.g., familial Mediterranean fever) caused by a dysfunction of the immune system can affect the innate immune system. The well-established association between COVID-19 and the development of cytokine release syndrome, as well as the triggering of autoimmunity support the hypothesis that COVID-19 may trigger autoimmune thyroid diseases, including autoimmune hypothyroidism [22]. However, dental care perspectives in these patients, similar to patients with other comorbidities, are yet to be clarified.

An ongoing study carried out in an Italian population aimed to systematically evaluate the prevalence and incidence of patients with congenital bleeding disorders who were also infected with COVID-19 [23]. A comparison of our findings on immune system disorders and hematological diseases with other published findings indicated a high prevalence of these diseases in the youngest age group [7, 14]. A relationship between autoinflammatory disorders with underlying genetic pathologies and certain hematological diseases of congenital origin may explain this outcome.

A history of COVID-19 infection was the fourth most common pre-existing condition in the present retrospective study with an incidence rate of 4.5%, sharing its rank with psychological disorders. The difference in the incidence of either of these disorders as a function of age or gender failed to reach statistical significance. The incidence of psychological disorders among dental patient populations has been evaluated [7-8]. In the current study, we found a high proportion of psychological disorders suggesting that social isolation during the pandemic may be a risk factor for the development of depressive symptoms.

Data on the proportion of respiratory diseases in the current cohort were similar to the data reported on dental school patients in Iran and the West Indies [8, 11]. Contrary to the current study, Bozdemir et al. reported COPD as the most common respiratory disease among dental patients in a study carried out prior to the SARS-CoV-2 pandemic [12]. Acute respiratory distress syndrome (ARDS) is a severe pulmonary disease, which is one of the major complications seen in COVID-19 patients. ARDS may emanate from a dysregulation of the immune system, as well as imbalances in cytokine release and immune cell activation [24].

Unlike the current work, several studies have reported GI disturbances and musculoskeletal diseases as common systemic diseases among dentistry patients [11-12, 15]. ACE2 is a functional cellular receptor for SARS-CoV-2. The oral cavity is at potential risk for SARS-CoV-2 infection, as the oral mucosa expresses ACE2 [1]. Similarly, interactions between SARS-CoV-2 and organs of the GI tract may occur via the ACE2, suggesting that the GIS is likely to be affected in COVID-19 [25].

Myalgia is one of the most common manifestations of COVID-19, appearing in nearly 36% of COVID-19 patients. Increased release of cytokines as well as elevation of clinical laboratory markers of inflammation in patients with COVID-19 point to the presence of a generalized inflammatory response, explaining the presence of myalgia [26]. Furthermore, ACE2 receptors are found in skeletal muscle and synovial tissue, suggesting that viral invasion of these tissues may contribute to the symptoms [27]. However, the low proportion of dental patients with GI and musculoskeletal diseases in the current study raises the question on whether the incidence of these diseases was exacerbated during the SARS-CoV-2 pandemic or whether the infection resulted in the onset of these diseases.

Many COVID-19 survivors experience persistent physical symptoms such as cough, fatigue, dyspnea, and pain after recovery [3]. Orofacial pain has been also reported in patients with chronic pain associated with SARS-CoV-2 infection, this may be a new area of research in dental medicine [26].

The pandemic has provided valuable lessons for the development of appropriate and relevant research infrastructure for human health. In the current study, we evaluated a comprehensive list of known risk factors for underlying medical conditions; these are well-established risk factors rather than those relevant to the specific circumstances of the pandemic. The findings of this study suggest several inferences. First, data on obesity was not recorded in the databases utilized in the current study. Only one study was carried out in a dental setting on patients with childhood obesity [28]. Therefore, it would be beneficial to develop a template to obtain a comprehensive medical history from patients by taking full account of the consequences of the pandemic. Second, comorbidities are common among dental patients; therefore, an interdisciplinary approach should be considered in providing dental care services to these patients. Larger prospective studies will help establish more definitive information on the interrelations between COVID-19 and other disorders, incorporating dental care perspectives.

Major adjustments in lifestyle and education had to be made during the SARS-CoV-2 pandemic. Knowledge of the incidence and determinants of common or rare medical conditions is fundamental for healthcare providers in order to organize appropriate prevention and treatment strategies at both individual and community levels. The type of diseases afflicting the population is evolving and the dental profession also needs to respond to these changes. Monitoring of the adult dental population with comorbidities can be used to obtain a more comprehensive list of underlying medical conditions in the population where appropriate. One option is taking a more exhaustive medical history of dental patients.

## Conclusions

Severe COVID-19 in adults has been linked to many different comorbidities. In this cross-sectional study of 1067 adult dental patients, 38.3% of patients with comorbidities sought dental treatment despite the ongoing pandemic. Hypertension and diabetes were the most frequent comorbidities whereas psychological diseases and recovery from SARS-CoV-2 infection were the fourth most common comorbidities of the current study population. The potential long-term effects of COVID-19 remain highly unpredictable and the data on outcomes in patients with different medical conditions are sparse. Dental schools should train dentists to help identify the more vulnerable patients in future pandemics.

## References

[1] Beyerstedt S, Casaro EB, Rangel ÉB (2021). COVID-19: angiotensin-converting enzyme 2 (ACE2) expression and tissue susceptibility to SARS-CoV-2 infection. Eur J Clin Microbiol Infect Dis.

[2] Alsaegh A, Belova E, Vasil'ev Y (2021). COVID-19 in dental settings: novel risk assessment approach. Int J Environ Res Public Health.

[3] Kompaniyets L, Pennington AF, Goodman AB (2021). Underlying medical conditions and severe illness among 540,667 adults hospitalized with COVID-19, March 2020-March 2021. Prev Chronic Dis.

[4] Smeets EC, de Jong KJ, Abraham-Inpijn L (1998). Detecting the medically compromised patient in dentistry by means of the medical risk-related history. A survey of 29,424 dental patients in The Netherlands. Prev Med.

[5] Jainkittivong A, Aneksuk V, Langlais RP (2004). Medical health and medication use in elderly dental patients. J Contemp Dent Pract.

[6] Ilguy M, Ilguy D, Dincer A (2005). The frequency of patients with cardiovascular and endocrine diseases in a dental faculty. OHDMBSC.

[7] Radfar L, Suresh L (2007). Medical profile of a dental school patient population. J Dent Educ.

[8] Bayaty HF, Murti PR, Naidu RS (2009). Medical problems among dental patients at the school of dentistry, the university of the West Indies. J Dent Educ.

[9] Dhanuthai K, Sappayatosok K, Bijaphala P (2009). Prevalence of medically compromised conditions in dental patients. Med Oral Patol Oral Cir Bucal.

[10] Bhateja S (2012). High prevalence of cardiovascular diseases among other medically compromised conditions in dental patients: a retrospective study. J Cardiovasc Dis Res.

[11] Maryam A, Atessa P, Mozafari Pegah M (2015). Medical risk assessment in patients referred to dental clinics, Mashhad, Iran (2011-2012). Open Dent J.

[12] Bozdemir E, Yilmaz HH, Orhan H (2016). General health and oral health status in elderly dental patients in Isparta, Turkey. East Mediterr Health J.

[13] Walia IS, Bhatia L, Singh A (2017). Prevalence of medical comorbidities in dental patients. Ann Int Med Den Res.

[14] Wadhwani RB, Tharani DA (2017). Prevalence of systemic diseases among patients attending department of dentistry at government medical college and hospital in Latur, India. MIJODEN.

[15] Taghibakhsh M, Moezzi Ghadim N, Rayatzadeh M (2018). Evaluation of the prevalence of systemic diseases in patients referring to the oral and maxillofacial medicine department of the Dental Branch of Islamic Azad University of Tehran during 2016-17. J Res Dent Maxillofac Sci.

[16] Frydrych AM, Parsons R, Kujan O (2020). Medical status of patients presenting for treatment at an Australian dental institute: a cross-sectional study. BMC Oral Health.

[17] Mueller AL, McNamara MS, Sinclair DA (2020). Why does COVID-19 disproportionately affect older people?. Aging (Albany NY).

[18] Ferrucci L, Fabbri E (2018). Inflammageing: chronic inflammation in ageing, cardiovascular disease, and frailty. Nat Rev Cardiol.

[19] Liu Y, Pan Y, Yin Y (2021). Association of dyslipidemia with the severity and mortality of coronavirus disease 2019 (COVID-19): a meta-analysis. Virol J.

[20] Fleming N, Sacks LJ, Pham CT (2021). An overview of COVID-19 in people with diabetes: pathophysiology and considerations in the inpatient setting. Diabet Med.

[21] Yazdanpanah N, Rezaei N (2022). Autoimmune complications of COVID-19. J Med Virol.

[22] Duntas LH, Jonklaas J (2021). COVID-19 and thyroid diseases: a bidirectional impact. J Endocr Soc.

[23] Coluccia A, Marchesini E, Giuffrida AC (2021). Addressing the impact of SARS-CoV-2 infection in persons with congenital bleeding disorders: the Italian MECCOVID-19 study. Haemophilia.

[24] Juanes-Velasco P, Landeira-Viñuela A, García-Vaquero ML (2022). SARS-CoV-2 infection triggers auto-immune response in ARDS. Front Immunol.

[25] Zhang H, Shao B, Dang Q (2021). Pathogenesis and mechanism of gastrointestinal infection with COVID-19. Front Immunol.

[26] Alonso-Matielo H, da Silva Oliveira VR, de Oliveira VT (2021). Pain in Covid Era. Front Physiol.

[27] Desai AD, Lavelle M, Boursiquot BC (2022). Long-term complications of COVID-19. Am J Physiol Cell Physiol.

[28] Greenberg BL, Glick M, Tavares M (2017). Addressing obesity in the dental setting: What can be learned from oral health care professionals' efforts to screen for medical conditions. J Public Health Dent.

